# Risk of Mortality (Including Sudden Cardiac Death) and Major Cardiovascular Events in Atypical and Typical Antipsychotic Users: A Study with the General Practice Research Database

**DOI:** 10.1155/2013/247486

**Published:** 2013-12-26

**Authors:** Tarita Murray-Thomas, Meghan E. Jones, Deven Patel, Elizabeth Brunner, Chetan C. Shatapathy, Stephen Motsko, Tjeerd P. Van Staa

**Affiliations:** ^1^Clinical Practice Research Datalink, Medicines and Healthcare Products Regulatory Agency, London SWIW 9SZ, UK; ^2^Eli Lilly and Company, Indianapolis, IN, USA; ^3^London School of Hygiene & Tropical Medicine, London, UK; ^4^Utrecht Institute for Pharmaceutical Sciences, Utrecht University, Utrecht, The Netherlands

## Abstract

*Objective*. Antipsychotics have been associated with increased cardiac events including mortality. This study assessed cardiac events including mortality among antipsychotic users relative to nonusers. *Methods*. The General Practice Research Database (GPRD) was used to identify antipsychotic users, matched general population controls, and psychiatric diseased nonusers. Outcomes included cardiac mortality, sudden cardiac death (SCD), all-cause mortality (excluding suicide), coronary heart disease (CHD), and ventricular arrhythmias (VA). Sensitivity analyses were conducted for age, dose, duration, antipsychotic type, and psychiatric disease. *Results*. 183,392 antipsychotic users (115,491 typical and 67,901 atypical), 544,726 general population controls, and 193,920 psychiatric nonusers were identified. Nonusers with schizophrenia, dementia, or bipolar disorder had increased risks of all-cause mortality compared to general population controls, while nonusers with major depression had comparable risks. Relative to psychiatric nonusers, the adjusted relative ratios (aRR) of all-cause mortality in antipsychotic users was 1.75 (95% CI: 1.64–1.87); cardiac mortality 1.72 (95% CI: 1.42–2.07); SCD primary definition 5.76 (95% CI: 2.90–11.45); SCD secondary definition 2.15 (95% CI: 1.64–2.81); CHD 1.16 (95% CI: 0.94–1.44); and VA 1.16 (95% CI: 1.02–1.31). aRRs of the various outcomes were lower for atypical versus typical antipsychotics (all-cause mortality 0.83 (95% CI: 0.80–0.85); cardiac mortality 0.89 (95% CI: 0.82–0.97); and SCD secondary definition 0.76 (95% CI: 0.55–1.04). *Conclusions*. Antipsychotic users had an increased risk of cardiac mortality, all-cause mortality, and SCD compared to a psychiatric nonuser cohort.

## 1. Introduction

Antipsychotics used for the treatment of schizophrenia and other mental illnesses have been associated with increased mortality (including sudden cardiac death) and major cardiac events. Several observational studies have assessed this association [1–4]. A retrospective cohort study of Medicaid enrollees in Tennessee found that for current users of both typical and atypical antipsychotics the risk of sudden cardiac death increased with increasing dose [4]. Among users of atypical agents, the incidence-rate ratios increased from 1.59 (95% CI, 1.03–2.46) for those taking low doses to 2.86 (95% CI, 2.25–3.65) for those taking high doses. Limitations of the study included the inability to fully control for major potential confounders (e.g., increasing disease severity with increasing dose, untreated psychosis, unhealthy lifestyle, current smoking, abuse of alcohol and other substances [4]), and the validity of the definition of sudden cardiac death (SCD).

The mechanism for the potential increased risk of sudden cardiac death with the use of antipsychotics is not known. A possible association between sudden cardiac death has been explained by a lengthening of ventricular repolarization (QT interval prolongation) predisposing to life-threatening ventricular tachyarrhythmias (i.e., torsades de pointes) [4]. However, it has been described that antipsychotics may have a clear differential risk in prolonging the QT interval [5]. More than 80% of cases of sudden cardiac death occur in individuals with coronary heart disease [6–8]. SCD occurs in only 5% to 10% of subjects without a positive history for coronary heart disease or congestive heart failure [9] and the most common electrophysiological mechanisms leading to SCD are ventricular tachyarrhythmias. Risk factors for SCD include family history of coronary artery disease, increased LDL cholesterol, hypertension, smoking, and diabetes mellitus [10–13]. Various reports suggest that patients with schizophrenia have a significantly increased burden of cardiovascular disease and diabetes than the general population and that cardiovascular mortality contributes to the excess mortality in persons with schizophrenia regardless of their treatment [2, 14–16]. This increased burden of metabolic changes could increase the risk of SCD.

To further evaluate the findings of these studies, this retrospective database study was performed to assess the primary the potential risk of cardiac mortality including sudden cardiac death as the primary outcome and the following secondary outcomes—the risk of all-cause mortality (excluding suicide) and of major cardiac events (including acute myocardial infarction (AMI), CHD, and life-threatening ventricular arrhythmias), in the antipsychotic exposed population. This study attempted to adjust for additional known risk factors for SCD not previously assessed in other studies, smoking status, body mass index, and alcohol status, and to address issues with the definition of sudden cardiac death. The study was designed to evaluate the potential relationship between antipsychotic drug use, psychiatric illness, cardiac mortality, all-cause mortality (excluding suicide), and major cardiac events.

## 2. Methods

### 2.1. Data Source

This study used data from the UK General Practice Research Database (GPRD), now administered by the Clinical Practice Research Datalink. GPRD is comprised of anonymised computerised medical records from primary care for about 8% of the UK population. GPs play a key role in the UK health care system, as they are responsible for primary health care and specialist referrals. Details on the history of GPRD and validation studies have been published [17, 18]. At the time of this study, about 40% of the practices participated in the anonymous and patient-level linkage to the national registry of hospital admission (Hospital Episode Statistics [HES]) and death certificates (as collected by the Office of National Statistics [ONS]). For each hospitalised patient, their hospital charts are reviewed and their dates of admission, discharge and main diagnoses are extracted, coded by coding staff, and collated nationally into HES. The death certificates list the date and causes of death. HES data were available from April 1997 and death certificates from January 2001. The data from HES, death certificates, and GPRD were recorded and collected independently from each other.

### 2.2. Study Population

#### 2.2.1. Antipsychotic Users

**Figure 1:**
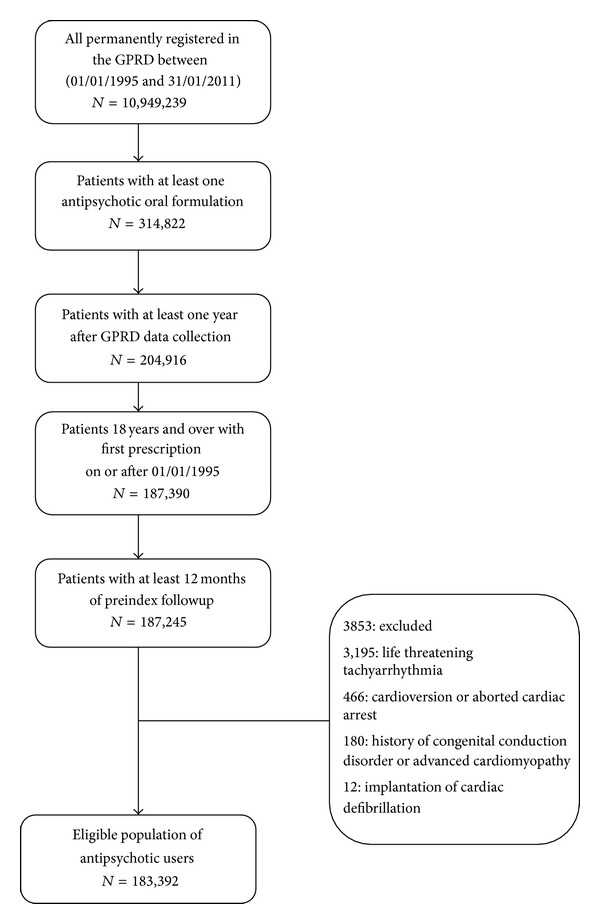
Flow diagram showing the composition of the study population, oral antipsychotic users.

A flow chart of the population of antipsychotic users (exposed cohort) eligible for study is shown in Figure 1. Antipsychotic medications screened in this study are listed in Table 1 and were identified on the basis of the corresponding Multilex codes for the respective therapy. Patients were classified at the index date (first date of antipsychotic prescribing in the study period) into prevalent and incident antipsychotic users: incident users as those with a first-ever prescription; prevalent users as those who received an antipsychotic prescription prior to index date. Each user of antipsychotics was matched to three general population controls on age, gender, general practice, and calendar time.

#### 2.2.2. General Population Controls

General population controls had no recorded use of antipsychotics (no recorded Multilex code for screened antipsychotics (Table 1)) or no history of psychiatric disorders (no documentation of a Read coded diagnosis for schizophrenia, bipolar disorder/other mood disorders, major depression, or dementia in the patient clinical or referral record). The index date was at least 12 months after the start of GPRD data collection for each patient.

#### 2.2.3. Psychiatric Nonusers

A cohort of patients with a diagnosis of schizophrenia, bipolar disorder/other mood disorders, major depression, or dementia but without a history of use of antipsychotics was also identified (referred to as psychiatric nonusers). The index date was defined as the date of the first record of the psychiatric disorder (i.e., both incident and prevalent cases were included).

Psychiatric non-users who were subsequently prescribed antipsychotics after the index date of their psychiatric disorder were censored at the date of their first antipsychotic prescription. Thus, person time for psychiatric nonusers consisted of follow-up between their first psychiatric diagnosis date and the end of follow-up (for patients with no record of antipsychotic treatment) or prior to their antipsychotic censor date for those who were treated. The duration of psychiatric disease was calculated as the difference between the earliest of the antipsychotic censor date, patient's transfer out date, or practice last collection date, and the date of first psychiatric disorder recorded during the GPRD followup. This cohort was not matched to the cohort of antipsychotic users; rather baseline differences were adjusted for using multivariate analysis.

#### 2.2.4. Exclusions

Patients with a Read coded medical diagnosis of a congenital conduction disorder or advanced cardiomyopathy (hypertrophic or dilated) at any time in the patient record were excluded. Patients with a Read coded medical diagnosis of life-threatening ventricular tachyarrhythmia, cardioversion, aborted cardiac arrest, or implantation of a cardiac defibrillator prior to or on their registration date were also excluded. Additionally, after matching (where applicable), patients with a life-threatening ventricular tachyarrhythmia, cardioversion, aborted cardiac arrest, or implantation of a cardiac defibrillator prior to or on the index date were excluded.

Patients were followed from the index date up to the occurrence of the outcome of interest or the end of data collection (i.e., last GPRD data collection and transfer out of the practice or date of death, whichever date came first).

### 2.3. Antipsychotic Treatment

#### 2.3.1. Treatment Exposure

For antipsychotic users, treatment exposure was classified into three periods: (i) “*current exposure*” is the period from issuing an antipsychotic prescription up to 1 month after the expected end of treatment. Patients who were issued repeat prescription within a month (30.4 days) after the expected end of a prescription were assumed to be continuously treated (ii) *recent exposure* is the period from 1 month after the expected end of use of a treatment until 6 months after, and (iii) *past exposure* is any subsequent follow-up time after recent treatment (until censoring or a further prescription). In some analyses current treatment was also examined according to cumulative duration of treatment or the strength of treatment at any given time period. Followup among users was restricted to the “current exposure” period.

The defined daily dose (DDD) for prescriptions was calculated based on the prescribed daily dose and strength per tablet. These DDDs were then converted to the chlorpromazine equivalents of low, medium, or high dose using the dose thresholds for antipsychotics defined in chlorpromazine equivalents [20]. Given the British National Formulary (BNF) recommendation that DDDs should be between 25 and 1000 mg, converted DDDs outside of this range were treated as unknown values. All unknown DDD values were imputed using the median value of the distribution of all calculable DDDs for the respective antipsychotic therapy and where this was unavailable, using the median value for individual drug types. The overall proportion of unknown DDD records which were imputed was 9%. Estimated DDDs converted to chlorpromazine equivalents per day were classified as low daily dose [<200 mg], medium [200–399 mg], and high [≥400 mg].

#### 2.3.2. Treatment Duration

The estimated duration of use of therapy was calculated as the prescription quantity divided by daily dose. Where the estimated duration was missing or was estimated as <1 or >91.4 days (corresponding approximately to the 1st and 99th percentiles of the distribution of all estimated prescription durations), the estimated durations were imputed using the median for that prescription, or where this was unavailable, using the median pertaining to the subtype of that substance instead. The overall proportion of estimated duration records, across all prescriptions, which were imputed, was 13%. The tertiles of the distribution of cumulative treatment duration at the end of followup for all exposed patients were calculated. These cutoffs corresponded broadly to (a) <1 year, (b) 1–3 years, or (c) >3 years and were used to further categorise current treatment for analyses investigating cumulative treatment duration.

### 2.4. Outcomes of Interest

#### 2.4.1. Primary and Secondary Outcomes

The outcomes of interest included cardiac mortality, three definitions for sudden cardiac death (SCD), all-cause mortality excluding suicide (referred to as all-cause mortality from here on out), coronary heart disease (CHD), and life-threatening ventricular arrhythmias. Outcomes were identified using GPRD Read codes, ICD10 codes from ONS death certificates data, and records from HES (Table 2). In addition, GPRD free text information was used to improve the odds of accurately identifying SCD cases.

#### 2.4.2. Free Text Search Methodology

The average number of characters of free text per patient was estimated for each practice in GPRD stratified by calendar time. Based on the changes in average amount of free text over calendar time, a starting year of 2006 was selected for use of free text. Practices were then ranked by the average amount of free text overall. The Free text review for primary and secondary SCD cases was restricted to the 243 practices with an above median amount of free text per patient in each practice and restricted to followup after 2006. The free texts in the 3 weeks around a death date were searched for string terms suggestive of sudden cardiac death (Table 2). Anonymised text before and after each instance that a text string appeared was selected, to a maximum of 500 characters (including spaces) before and 500 characters after the string. Free text results were then independently reviewed by two internal clinical experts from Lilly (a third expert was used in case of discordant assessment) and relevant events were included.

#### 2.4.3. Case Ascertainment Using GPRD Read Codes, Free Text, and Death Certificate Data

When querying multiple sources for the occurrence of an outcome, the following hierarchy was used, with earlier-named sources taking precedence over the later-named sources when more than one was available for any given patient: death certificate (both the recorded date and cause of death), followed by GPRD free text (date of death taken to be the minimum of the recorded GPRD date of death or free text date), followed by GPRD Read code (date of death taken to be the minimum of the recorded GPRD date of death or the date of Read code). Where both a Read code and a free text term were recorded, the earlier of the two events was considered as the date of the event. For example, under our primary definition of sudden cardiac death a patient was classified as a case on the basis of their cause of death but not on their GPRD Read codes only. However, if their caseness was based on GPRD Read codes there was no need for supporting evidence in the free text also. A conservative approach in identifying the SCD cases was used, counting them as SCD cases unless the free text clearly specified another cause of death.

### 2.5. Statistical Analyses

Poisson regression analysis was used to examine the rates of each outcome and produce corresponding age and sex-adjusted (RRs) and fully adjusted relative ratios (aRRs) and 95% confidence intervals (CI) for each comparison. Adjusted models were fitted using automated backward elimination with stepwise regression. To ensure that the resulting models were not overparameterised, the number of variables to be included in the model was selected using the rule of thumb of five outcome events per parameter [21, 22]. Goodness-of-fit was assessed by examining the associated *P* values of the covariates such that a covariate was included in the model only if the associated *P* value was <0.05, while any covariates in the model with *P* values ≥0.10 were excluded. The following patient characteristics were considered as potential confounders in regression models: age, sex, socioeconomic status (Index of Multiple Deprivation calculated at the postcode of the patient's residence), smoking history, alcohol use, and body mass index. Age, sex, and treatment (atypical, typical, or current antipsychotic use, depending on analysis) were included in the model a priori. BMI was calculated using the most recent BMI measurement available prior to index date. Nonsmokers were classified as patients with a record of nonsmoking only, documented in their record. Current smoking was based on the most recent smoking record of the patient documented prior to the index date. Regression models included indicator variables for missing values for BMI and smoking. Other confounding factors included were time since start of GPRD data collection (in tertiles), duration of psychiatric disease, history of cardiovascular disease, alcohol or drug abuse, diabetes mellitus, history of suicide attempt, prior hospital admission for psychiatric disease and prescribing in the 3 months before of statins or fibrates, antihypertensive drugs, warfarin, antiplatelets, nitrates, lithium, antiepileptics, antidepressants, and anxiolytics. To examine changes in risk over time, patient followup (from start of medication until end of data collection) was subdivided into 100 periods of equal length and incidence rates (hazard rates) estimated for each period. These rates were smoothed using the methods proposed by Ramlau-Hansen [23]. Analyses were conducted using STATA 11.

## 3. Results

The exposed cohort included 183,392 antipsychotic users (Table 3). Patients had a mean age of 60.3 years (SD: 22.1) and average duration of follow-up of 4.0 years (SD: 4.0). Typical antipsychotic users (115,491 (63.0%)) were slightly older (mean age: 61.4 years (SD: 21.1)) compared to atypical users (mean age: 58.3 years (SD: 23.3)) and were followed for a longer duration than atypical users (4.7 years (SD: 4.5) versus 2.9 years (SD: 2.7)). Typical antipsychotic users also had a longer mean duration of psychiatric disease (6.8 years (SD: 9.8)) when compared to atypical users (5.8 years (SD: 8.8)). Specific GPRD codes for the type of psychiatric disease were not available in two-thirds of the exposed cohort due to lack of specific psychiatric diagnosis documented in the patient's record. Patients in the exposed cohort were well matched to general population controls on age, gender, general practice and calendar time. The mean age and duration of follow-up of general population controls was 60.0 years (SD: 21.8) and 4.8 years (SD: 4.0), respectively. The psychiatric nonuser cohort comprised of 193,920 patients with a mean duration of follow-up of 4.1 years (SD: 3.5). Psychiatric nonusers were on average younger (52.3 years (SD: 22.0)) and had a shorter duration of psychiatric disease (0.9 years (SD: 4.1)) compared to the exposed cohort.

The use of typical antipsychotics decreased over calendar time, while that of atypical antipsychotics increased. The mean cumulative duration of prescribing among patients initiated on antipsychotics was 1.6 years (SD: 2.7). The mean cumulative duration of prescribing of typical and atypical antipsychotics during the study period was 1.2 years (SD: 2.4) and 2.1 years (SD: 2.9), respectively, regardless of the type of antipsychotic initiated at cohort entry.

Table 4 shows the results of the comparisons of the psychiatric nonuser cohort and general population controls, providing an analysis of the possible extent of confounding. In the psychiatric nonuser cohort, compared to the general population, age and sex-adjusted rates of all-cause mortality were increased in patients with schizophrenia, dementia, bipolar disorder, and major depression. This trend persisted after adjustment for major risk factors of all-cause mortality among conditions of interest except for major depression. However, the possibility of confounding by unmeasured risk factors such as disease severity cannot be excluded. There were also trends for increased risks of cardiac mortality in unexposed patients with major affective disorders compared to the general population, although the number of cases was smaller which did not allow for fully adjusted comparisons.

The rates of cardiac mortality, all-cause mortality, and sudden cardiac death (regardless of the definition) were increased in the exposed cohort compared to both the general population controls and the psychiatric nonuser cohort (Table 5). The risk of sudden cardiac death using the primary definition was increased fourfold in antipsychotic users (aRR of 4.03 (95% CI 2.63–6.16) compared to general population controls). There were no significant differences in the rate of CHD between the exposed cohort compared to the general population controls and the psychiatric nonuser cohort compared to their general population controls.

**Figure 2:**
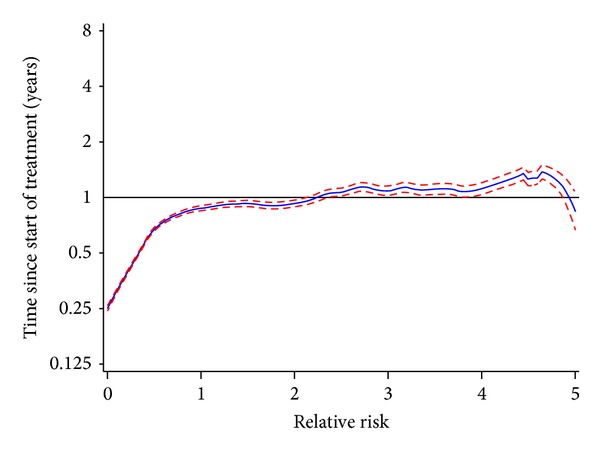
Smoothed crude RR of all-cause mortality (excluding suicide) and 95% Confidence Interval over treatment time in users of atypical antipsychotics compared to matched users of typical antipsychotics. *x*-axis: RR, *y*-axis: Time since start treatment (years).

The rates of all-cause mortality, cardiac mortality, SCD (using the tertiary definition), and CHD were significantly lower with atypical antipsychotics compared to typical antipsychotics, as shown in Table 6. The rate of sudden cardiac death (using the secondary definition) was 24% lower with atypical antipsychotics (aRR of 0.76 (95% CI 0.55–1.04)).  Figure 2 shows the crude ratios of smoothed hazard ratios of all-cause mortality in users of atypical antipsychotics compared to those in users of typical antipsychotics by year of treatment. The rate of all-cause mortality was lower with atypical antipsychotics during the first year of treatment while the rates were more comparable to those with typical antipsychotics with longer use.

Table 7 shows the primary causes of death in users of typical and atypical antipsychotics. The rate of death due to neoplasms was higher in users of typical antipsychotics, while death due to cardiovascular disease were similar to those patients using atypical antipsychotics.

The aRRs of all-cause mortality and cardiac mortality were found to vary by age (Table 8). The aRR of all-cause mortality was 4.12 in antipsychotic users aged <30 years and 1.43 in users aged ≥80 years. The incidence rates were considerably higher in elderly users, indicating that the excess absolute risks were largest in the elderly users. Increasing risk with increasing dose was observed among antipsychotic users for cardiac mortality, but not for all-cause mortality compared to psychiatric nonuser controls.

## 4. Discussion

This study used data from a large primary care population in the UK. We found that all-cause mortality and cardiac mortality (including SCD) were significantly higher among antipsychotic users versus controls. We also observed that the risk of all-cause mortality and cardiac mortality was lower when comparing current antipsychotic users to psychiatric non-users than when comparing current user to the general population. This finding suggests that the mortality risk for current antipsychotic users and psychiatric non-users is more similar than to that of the general population, suggesting an increased risk for severe psychiatric disorders irrespective of management. When the risks of cardiac mortality, all-cause mortality, and cardiovascular outcomes were assessed according to the first type of antipsychotic prescribed to users, the relatives rates of these outcomes were found to be lower in patients exposed to atypical compared to typical antipsychotics. An increased risk of all-cause mortality was found in psychiatric non-users with schizophrenia, dementia, bipolar disorder, or major depression, when compared to the general population controls. Statistical adjustment for baseline risk factors did not modify these results, except with major depression. Confounding by indication is a major challenge in this type of research. Patients diagnosed with psychiatric disorders who experience severe symptoms may be more likely to be treated with certain types of antipsychotic medication. Thus, if severity of disease is associated with the outcome under study, findings from observational studies may be a reflection of this unmeasured confounding rather than a true drug-disease relationship.

Patients with mental illness may be at higher risk of cardiovascular outcomes. The results of the mortality comparison between psychiatric nonuser patients and general population controls support the presence of substantial confounding in patients with psychiatric disease. Increased risks of mortality have also been reported in a large Danish study comparing patients admitted to a psychiatric hospital to the general population [24]. In the present study, statistical adjustment in regression analyses (beyond age and sex adjustment) did not minimise this confounding (i.e., the crude and adjusted effect estimates did not vary greatly), with the exception of major depression. More sophisticated techniques such as matching by propensity scores could be used, but this technique also cannot address confounding by unmeasured characteristics (such as clinical severity of psychiatric disease). It would clearly be preferable to evaluate the mortality risks of antipsychotics in a large randomised trial. Unfortunately, trials evaluating antipsychotics use are generally short in duration [25].

Although we found that the rates of CHD between the exposed cohort compared to general population controls and the diseased unexposed cohort were similar this observation may be partly explained by the very narrow definition used to identify CHD in the study—CHD was evaluated as a record of acute myocardial infarction (ICD-10 codes I21and I22) or cardiac procedures documented during the study period. It is also plausible that precursors of CHD go undiagnosed/undetected in psychiatric patients due to complexity of care and competing priorities in disease management.

The Food and Drug Administration has warned that the use of antipsychotics poses an increased risk of death in elderly patients with dementia-related psychosis. In a meta-analysis by Schneider et al. of randomized placebo-controlled parallel group clinical trials in patients with dementia, authors found that death occurred more often in subjects randomized to drug (3.5%) than to placebo (2.3%) [26]. We found that the risks of mortality and cardiovascular outcomes were lower with atypical compared to typical antipsychotics. Differences in baseline characteristics generally appeared smaller between users of typical and atypical antipsychotics. Our findings of lower mortality risks with atypical antipsychotics are consistent with other observational studies [27–31], with the exception of a small study in the Netherlands [32]. However, when the analysis was confined to users who were prescribed antipsychotic medication within 6 months of death the analysis of users of typical antipsychotics had a higher rate of death due to neoplasms but similar rates of death due to cardiovascular disease compared to users of atypical antipsychotics. This observation is likely confounded by disease duration and severity but may suggest that our finding of a more favourable outcome during treatment with atypical antipsychotics may be one that is apparent in the early stages of disease, but that long term use of atypical antipsychotics carries a similar risk of death to that of typical antipsychotics users. Further study is warranted.

A recent study by Huybrechts et al. of 75,000 new antipsychotic users found increased risks of death due to cardiovascular, cerebrovascular and respiratory disease in users of haloperidol compared to users of risperidone [30]. The results on death due to cancer were not presented and it is unknown whether the differential risks of mortality between typical and atypical antipsychotics also applied to death due to cancer. The mechanisms through which typical antipsychotics might increase the risk of death are unclear [31].

We compared our results to those of previous observational studies, most notably Ray et al. [4]. Consistent with Ray et al., when assessing SCD overall (using the secondary definition, which most closely corresponds to the Ray definition [4]), users of both typical and atypical antipsychotics had an increased risk of SCD when compared to nonusers with psychiatric illness. The risk of SCD increased with increasing atypical antipsychotic dose, a finding which was not borne out in the present study, although we did find increasing risk with increased doses of typical antipsychotics for the secondary SCD definition (results not shown). Our analysis assessing the risk of all-cause mortality among users of any antipsychotic relative to psychiatric nonusers (1.75 (95% CI, 1.64–1.87)) is consistent with that reported by Enger et al. (2.18 (95% CI, 1.14–4.18)) [2] in his study comparing the risk of all-cause mortality (excluding suicide), cardiovascular mortality, acute myocardial infarction (MI), and arrhythmias in people with schizophrenia who were prescribed antipsychotics. Reports by Osborn et al. [14] on a previous study conducted in the GPRD on the risk of cardiac and cancer mortality in people with severe mental illness also corroborate our findings of an increased relative risk of cardiac death among antipsychotic users.

We found some evidence of a direct dose-response relationship between antipsychotic prescribing and cardiac mortality. This relationship persisted even when antipsychotic therapy was stratified by typical and atypical use and individual antipsychotic therapy (results not shown). A similar trend was reported by Bralet et al. [33] albeit among male patients only, while Enger et al. [2] reported an inverse relationship. Ray et al. [4] reported a direct relationship between atypical antipsychotics and SCD, but our study was not sufficiently powered to provide reliable estimates of this effect. Using our primary definition of SCD we were only able to identify a very small number of cases with SCD (4 cases were prescribed medium dose and 1 case at high dose). This makes it difficult to draw any conclusions regarding a potential dose-response relationship with SCD.

Our finding of excess mortality across all age groups of psychiatric patients has been well described [34–37]. However, the decline in the aRR of all-cause and cardiac mortality with increasing age is surprising. As patients with psychiatric disease tend to have many risk factors for death, some of which could not be accounted for in our analysis, we expected to observe an increase in the relative risk of mortality with age. The age related decline in mortality may be explained by the healthy survivor effect [14, 38]; younger patients with poorer health and more serious psychiatric disease die earlier leaving the older and potentially healthier patients in the study. Our findings of an inverse age mortality trend are similar to that reported by Osborn et al. [14] who showed that the hazard ratio for CHD mortality in patients with severe mental illness relative to controls were 3.22 (95% CI, 1.99–5.21); 1.86 (95% CI, 1.63–2.12); and 1.05 (95% CI, 0.92–1.19) for people aged 18–49, 50–75, and >75, respectively.

The risk of mortality among users of atypical medications relative to typical users increased over time and was higher after two years of use. This increase in mortality may potentially be due to the longer-term outcomes in patients who experience changes in metabolic parameters during treatment with atypical antipsychotics [39] and to the growing problem of antipsychotic polypharmacy [40]. These metabolic factors could explain increased mortality over time and after two years of use; mortality secondary to potential changes in QT interval prolongation is likely to occur at earlier stages of treatment [5].

There are various strengths to this study. The present study included a large number of patients and outcomes that were obtained through three independently collected data sources, including prospectively collected hospital admission data and death certificates. Additionally, patient followup occurred over a relatively long period. Despite the study size and the availability of data from the real world setting, the findings of this study are limited by a number of factors.

In contrast to national trends in antipsychotic prescribing in England during the period 1998–2010 [41] and clinical guidelines from the National Institute of Clinical Excellence (NICE) [42] on prescribing for schizophrenia, conventional antipsychotics were more commonly prescribed as the initiating treatment in our study. These comparisons suggest that the exposed population in this study may not be representative of more current antipsychotic prescribing practices in the community. We are nonetheless confident that the exposed population was reflective of actual clinical practice as our observations are supported by a previous study undertaken in the GPRD [43]. King et al. reported a similar distribution of antipsychotic prescribing shortly after the introduction of atypical antipsychotics in the UK and found that older patients with schizophrenia and schizoaffective disorder were significantly less likely to be prescribed atypical antipsychotics relative to conventional antipsychotics. According to this study a five-year increase in age was associated with a 15% decrease in the probability of being prescribed an atypical antipsychotic. Given that atypical antipsychotics were just being introduced during the earlier parts of our study, that there were low periods of uptake during the study period, that the average age of our exposed population was about 60 years, and NICE recommendations on the prescribing of atypical antipsychotics only became available during 2002/2003, we feel that the distribution of prescribing observed in this study is plausible and representative of antipsychotic prescribing during the study period.

Due to the lack of information on important confounding factors such as the underlying severity of psychiatric disease or metabolic changes associated with antipsychotic treatment, these factors could not be addressed in this study. Psychiatric disease severity is also difficult to record; over sixty percent of the exposed cohort did not have a clinical or referral record for one of the four psychiatric diseases considered. This may be partly explained by the fact that more specific mental health diagnoses are likely to be made in secondary care or in the community mental health setting and that this information is not adequately communicated back to the general practice. Another potential explanation is the stigma associated mental health disease and the effect of documenting this in the patient record. Another limitation is that the exposure information was based on prescription information rather than by actual use. In addition, selection bias is also possible given that patients with ventricular arrhythmias (or any other underlying know cardiac condition) may lead to a higher rate of monitoring by ECGs and higher probability that the physician may record this outcome.

The definition of sudden cardiac death was restricted by the limited clinical detail in the GPRD records and the lack of precise timing of the sequence of events that lead to an out of hospital death. Moreover, death certificate data may not be a reliable means of identifying sudden cardiac death. We applied different definitions of SCD as a sensitivity analysis but the secondary and tertiary SCD definitions may have been broad. In an effort to overcome known limitations of identifying SCD through use of electronic medical record codes, a free text search strategy was used. However, this approach was still limited, as many cases had very little free text. Most commonly, “sudden death” or “died suddenly” was the only free text available. When more information was available, it was often determined that the patient did not experience SCD (e.g., deaths due to motor vehicle accident, drug overdose). The limited amount of information in many of the cases likely led to a high number of false positives. Other research has experienced the same challenges in defining SCD using electronic medical records [44].

In relation to the causes of SCD, it has long been established that antiarrhythmic drugs are capable of precipitating ventricular tachyarrhythmias and sudden cardiac death [45, 46]. It is therefore plausible that coprescription of antidepressants [47–50], anti-infective agents [51], or other arrhythmogenic drugs such as antihistamines [52, 53] may explain the rates of all-cause mortality and SCD observed in this study. The contribution of these factors to the risk of SCD was not explored in this study.

Due to the dynamic nature of the GPRD, information relating to important factors such as the date of onset and diagnosis of psychiatric disorders are not always known or accurately reported. This can have implications for ascertaining disease duration and severity. In our analysis of the psychiatric non-user population, the reported duration of psychiatric disease is underestimated given what is known about the age of onset of psychiatric disorders—onset can occur in adolescence for conditions such as schizophrenia or arise in late adulthood for conditions such as dementia. Lack of information to correctly classify disease duration could potentially result in a heterogeneous comparison group; one with varying propensities to be prescribed antipsychotic therapies, differing clinical characteristics and risk factors for death. The inability to adjust for confounding of this nature in the database setting means that estimates of the risk of all-cause and cardiac mortality among the psychiatric non-user population should be regarded with caution.

Though several limitations are acknowledged, we believe our findings contribute to the understanding of the potential cardiac-related risks with antipsychotic exposure in patients with severe mental illnesses.

## 5. Conclusions

Current users of antipsychotics had an increased risk of all-cause mortality, cardiac mortality, and risk of sudden cardiac death when compared to both the general population controls and the psychiatric nonuser cohort. However, the risk of developing CHD and ventricular arrhythmias did not differ significantly from the psychiatric nonusers. Psychiatric nonusers with a diagnosis of bipolar disorder, schizophrenia, and dementia had an increased risk of all-cause mortality relative to the general population. Rates of all-cause mortality, cardiac mortality, SCD (using the tertiary definition), and CHD were significantly lower among users of atypical antipsychotics compared to typical antipsychotic users. Both all-cause mortality and cardiac mortality were higher among antipsychotic users versus psychiatric nonusers. There is no clear driver to explain this association. An increased risk of all-cause mortality was seen in nonantipsychotic users with schizophrenia, dementia, or bipolar disorder compared with general population controls.

## Figures and Tables

**Table 1:** Antipsychotic drugs screened among the target population.

Antipsychotic medication	Drug substance
Atypical	Amisulpride
	Aripiprazole
	Clozapine
	Olanzapine
	Paliperidone
	Quetiapine
	Risperidone
	Sertindole
	Zotepine

Typical	Perphenazine
	Benperidol
	Chlorpromazine
	Chlorprothixene
	Droperidol
	Flupentixol
	Fluphenazine
	Haloperidol
	Levomepromazine
	Loxapine
	Oxypertine
	Pericyazine
	Pimozide
	Promazine
	Remoxipride
	Sulpiride
	Thioridazine
	Trifluoperazine
	Trifluperidol
	Zuclopenthixol

**Table 2:** Definitions for outcomes of interest.

All-cause mortality	Deaths due to any cause except suicide.
Cardiac mortality	Based on death certificates with ICD 10 codes I10, I11.9, I20, I21, I22, I23, I24, I25, I42.8, I42.9, I46, I47, I49.0, I49.8, I49.9, I51.6, I51.9, I70.9, R09.2, R96, R98

SCD primary definition	This was a narrow definition of SCD and the most restrictive. The SCD diagnosis was derived from the following ICD10 codes recorded on death certificates (available for January 1998 to November 2010): I46, I47.2, I49.0, R09.2, R96 [19], from Read codes in GPRD that correspond to these ICD10 codes, or from free text in the ±3 weeks before or after the death recording. Data from a national registry of hospital admission (Hospital Episode Statistics [HES], available for April 1997 to October 2010) were used to ensure that the event took place outside the hospital. A mortality event that occurred within 30 days after the hospital discharge date was not included in the SCD definition.

SCD secondary definition	This definition is broader than the primary definition and is similar to the definition used by Ray et al. (2009) [4]. The SCD diagnosis was derived from death certificates with ICD 10 codes I10, I11.9, I20, I21, I22, I23, I24, I25, I42.8, I42.9, I46, I47, I49.0, I49.8, I49.9, I51.6, I51.9, I70.9, R09.2, R96.1, R98, from Read codes in GPRD that correspond to these ICD10 codes, or from free text in the ±3 weeks before or after the death recording. Cases with a HES record of hospitalisation were excluded.

SCD tertiary definition	This definition was the same as the secondary definition with the exception that only data from GPRD and not from death certificates or HES were used.

SCD (based on free text)	The following text strings were used to identify sudden cardiac death from GPRD free text: “dropped dead,” “died unexpectedly,” “sudden cardiac death,” “death” and “cause unknown,” “acute cardiac death,” “unexpected” and “death,” “mors subita,” “death instanta,” “died instanta,” “sudden death,” “dropped dead,” and “died suddenly.”

CHD	Based on GPRD Read codes; data from a national registry of hospital admission (Hospital Episode Statistics (HES) ICD10 codes I21 or I22; or cardiac procedures)

Life-threatening Ventricular arrhythmias	Based on GPRD Read codes or HES ICD10 I49.0.

**Table 3:** Baseline characteristics at index date of the patients exposed to antipsychotics, psychiatric nonuser cohort, and general population controls.

Characteristic	Exposed to antipsychotics (*N* = 183,392)	Typical antipsychotics (*N* = 115,491)	Atypical antipsychotics (*N* = 67,901)	Psychiatric nonusers (*N* = 193,920)	General population control (*N* = 544,726)
Prevalent users	67,396 (36.7%)	40,258 (34.9%)	27,138 (40.0%)	0 (0.0%)	0 (0.0%)

Female	107,226 (58.5%)	69,426 (60.1%)	37,800 (55.7%)	132,458 (68.3%)	317,152 (58.2%)
Male	76,166 (41.5%)	46,065 (39.9%)	30,101 (44.3%)	61,462 (31.7%)	227,574 (41.8%)
Age: mean (sd)	60 (22)	61 (21)	58 (23)	52 (22)	60 (22)
Age: median (IQR)	62 (41–80)	64 (44–80)	58 (38–81)	48 (33–75)	61 (41–80)
Age 18–29	18,474 (10.1%)	9,180 (7.9%)	9,294 (13.7%)	34,559 (17.8%)	55,188 (10.1%)
Age 30–39	23,484 (12.8%)	13,747 (11.9%)	9,737 (14.3%)	38,574 (19.9%)	70,462 (12.9%)
Age 40–49	23,793 (13.0%)	14,554 (12.6%)	9,239 (13.6%)	28,555 (14.7%)	71,370 (13.1%)
Age 50–59	21,244 (11.6%)	14,627 (12.7%)	6,617 (9.7%)	21,302 (11.0%)	63,724 (11.7%)
Age 60–69	19,923 (10.9%)	14,461 (12.5%)	5,462 (8.0%)	13,831 (7.1%)	59,723 (11.0%)
Age 70–79	28,088 (15.3%)	18,881 (16.3%)	9,207 (13.6%)	21,194 (10.9%)	84,519 (15.5%)
Age 80+	48,386 (26.4%)	30,041 (26.0%)	18,345 (27.0%)	35,905 (18.5%)	139,740 (25.7%)
BMI: mean (sd)	26 (6)	26 (5)	26 (6)	26 (6)	26 (5)
Nonsmoker	69,482 (37.9%)	44,035 (38.1%)	25,447 (37.5%)	80,061 (41.3%)	249,362 (45.8%)
Ex-smoker	29,719 (16.2%)	17,638 (15.3%)	12,081 (17.8%)	39,550 (20.4%)	99,240 (18.2%)
Smoker	51,938 (28.3%)	31,361 (27.2%)	20,577 (30.3%)	57,004 (29.4%)	102,455 (18.8%)
Unknown smoking status	32,253 (17.6%)	22,457 (19.4%)	9,796 (14.4%)	17,305 (8.9%)	93,669 (17.2%)
Schizophrenia	15,475 (8.4%)	6,746 (5.8%)	8,729 (12.9%)	7,779 (4.0%)	0 (0.0%)
Bipolar disorder/other mood disorders	7,368 (4.0%)	3,170 (2.7%)	4,198 (6.2%)	4,700 (2.4%)	0 (0.0%)
Major depression	19,981 (10.9%)	11,680 (10.1%)	8,301 (12.2%)	134,105 (69.2%)	0 (0.0%)
Dementia	25,174 (13.7%)	12,669 (11.0%)	12,505 (18.4%)	47,336 (24.4%)	0 (0.0%)
No recorded specific psychiatric disorders	115,394 (62.9%)	81,226 (70.3%)	34,168 (50.3%)	0 (0.0%)	544,726 (100.0%)
History of alcoholism or drug abuse	19,933 (10.9%)	11,429 (9.9%)	8,504 (12.5%)	12,572 (6.5%)	13,256 (2.4%)
History of suicide attempts	10,777 (5.9%)	5,762 (5.0%)	5,015 (7.4%)	8,841 (4.6%)	3,537 (0.6%)
Diabetes mellitus	15,276 (8.3%)	9,480 (8.2%)	5,796 (8.5%)	13,639 (7.0%)	36,069 (6.6%)
Acute myocardial infarction	6,530 (3.6%)	4,224 (3.7%)	2,306 (3.4%)	5,466 (2.8%)	19,391 (3.6%)
Prescribing of statins/fibrates in the 3 months before	16,203 (8.8%)	8,406 (7.3%)	7,797 (11.5%)	18,124 (9.3%)	49,148 (9.0%)
Antiplatelets	32,260 (17.6%)	18,900 (16.4%)	13,360 (19.7%)	25,397 (13.1%)	72,622 (13.3%)
Antidepressants	83,595 (45.6%)	49,545 (42.9%)	34,050 (50.1%)	126,688 (65.3%)	34,393 (6.3%)
Anxiolytics	27,389 (14.9%)	15,637 (13.5%)	11,752 (17.3%)	12,907 (6.7%)	10,185 (1.9%)
Selective serotonin reuptake inhibitors	44,428 (24.2%)	24,330 (21.1%)	20,098 (29.6%)	98,373 (50.7%)	16,263 (3.0%)
Lithium	5,122 (2.8%)	2,851 (2.5%)	2,271 (3.3%)	1,743 (0.9%)	115 (<0.1%)
Antiepileptics	10,090 (5.5%)	5,853 (5.1%)	4,237 (6.2%)	3,817 (2.0%)	5,855 (1.1%)

**Table 4:** Confounding by underlying psychiatric disease comparing rates of all-cause mortality (excluding suicide) or cardiac mortality in the psychiatric nonuser cohort to that of the general population controls.

Outcome	Psychiatric Disease	No. of cases	Incidence rate per 1000 person-years	Age, sex-adjusted relative risk (RR) (95% CI)	Fully adjusted RR (aRR) (95% CI)
Mortality	None (general population)	295	10.9	Reference	Reference
	Patients with bipolar disorders	43	15.2	1.50 (1.09–2.07)^†^	1.40 (1.01–1.95)^†^
	None (general population)	19,858	96.5	Reference	Reference
	Dementia	4,445	142.8	1.54 (1.49–1.59)^‡^	1.45 (1.40–1.50)^‡^
	None (general population)	8,890	9.9	Reference	Reference
	Major depression	3,230	11.3	1.40 (1.35–1.46)^‡^	1.05 (0.98–1.12)
	None (general population)	513	10.1	Reference	Reference
	Schizophrenia	55	23.1	2.23 (1.69–2.94)^‡^	1.99 (1.50–2.65)^‡^

Cardiac mortality	None (general population)	34	1.8	Reference	Reference
	Patients with bipolar disorders	5	2.4	—(NC^#^)	—(NC^#^)
	None (general population)	4257	28.9	Reference	Reference
	Dementia	795	32.2	1.16 (1.08–1.25)^‡^	1.15 (1.06–1.24)^‡^
	None (general population)	1363	2.4	Reference	Reference
	Major depression	484	2.7	1.39 (1.25–1.54)^‡^	1.00 (0.85–1.17)
	None (general population)	66	2.2	Reference	Reference
	Schizophrenia	8	5.5	2.04 (0.98–4.26)*	—(NC^#^)

*P < 0.10, ^†^ *P* < 0.05, ^‡^ *P* < 0.01, ^#^Not calculable due to small number of cases.

**Table 5:** RR of all-cause mortality (excluding suicide) and cardiac mortality in exposed cohort, psychiatric nonuser cohort, and general population controls.

Outcome	Exposure	No. of cases	Incidence rate per 1000 person-years	Age, sex-adjusted relative risk (RR) (95% CI)	Fully adjusted RR (aRR) (95% CI)
All-cause mortality	General population	41,546	39.6	Reference	Reference
	Current use	23,841	86.1	2.98 (2.93–3.03)^‡^	2.72 (2.67–2.77)^‡^
	Psychiatric nonusers	7,765	25.3	Reference	Reference
	Current use	23,841	86.1	2.15 (2.10–2.21)^‡^	1.75 (1.64–1.87)^‡^

Cardiac mortality	General population	6,532	11.9	Reference	Reference
	Current use	2,478	17.0	2.01 (1.92–2.10)^‡^	1.83 (1.74–1.93)^‡^
	Psychiatric nonusers	1,289	6.4	Reference	Reference
	Current use	2,478	17.0	1.62 (1.52–1.74)^‡^	1.72 (1.42–2.07)^‡^

SCD-primary definition	General population	46	0.8	Reference	Reference
	Current use	44	2.7	4.45 (2.94–6.73)^‡^	4.03 (2.63–6.16)^‡^
	Psychiatric nonusers	10	0.4	Reference	Reference
	Current use	44	2.7	5.76 (2.90–11.45)^‡^	5.76 (2.90–11.45)^‡^

SCD-secondary definition	General population	244	4.0	Reference	Reference
	Current use	178	11.0	3.48 (2.87–4.23)^‡^	2.89 (2.32–3.60)^‡^
	Psychiatric nonusers	98	4.1	Reference	Reference
	Current use	178	11.0	2.36 (1.84–3.02)^‡^	2.15 (1.64–2.81)^‡^

SCD-tertiary definition	General population	370	2.3	Reference	Reference
	Current use	198	4.6	2.47 (2.08–2.94)^‡^	2.11 (1.73–2.56)^‡^
	Psychiatric nonusers	119	1.8	Reference	Reference
	Current use	198	4.6	1.92 (1.53–2.42)^‡^	1.79 (1.42–2.27)^‡^

CHD	General population	5,993	6.6	Reference	Reference
	Current use	1,269	5.4	1.06 (1.00–1.13)*	0.94 (0.87–1.00)*
	Psychiatric nonusers	982	3.5	Reference	Reference
	Current use	1,355	5.7	1.08 (1.00–1.18)*	1.16 (0.94–1.44)

Ventricular arrhythmias	General population	1,977	2.2	Reference	Reference
	Current use	646	2.7	1.53 (1.40–1.67)^‡^	1.49 (1.35–1.64)^‡^
	Psychiatric nonusers	500	1.8	Reference	Reference
	Current use	646	2.7	1.14 (1.01–1.28)^†^	1.16 (1.02–1.31)^†^

*P < 0.10, ^†^ *P* < 0.05, ^‡^ *P* < 0.01.

**Table 6:** RR of all-cause mortality (excluding suicide) or cardiac mortality in users of atypical and typical antipsychotics.

Outcome	Type of antipsychotic	No. of cases	Incidence rate per 1000 person-years	Age, sex-adjusted relative risk (RR) (95% CI)	Fully adjusted RR (aRR) (95% CI)
All-cause mortality	Typical	15,334	91.5	Reference	Reference
	Atypical	7,555	76.0	0.79 (0.77–0.81)^‡^	0.83 (0.80–0.85)^‡^
Cardiac mortality	Typical	1,130	17.8	Reference	Reference
	Atypical	1,214	16.1	0.84 (0.78–0.91)^‡^	0.89 (0.82–0.97)^‡^
SCD primary definition	Typical	20	3.8	Reference	Reference
	Atypical	22	2.1	—(NC^#^)	—(NC^#^)
SCD secondary definition	Typical	77	14.5	Reference	Reference
	Atypical	95	9.1	0.72 (0.53–0.97)^†^	0.76 (0.55–1.04)*
SCD tertiary definition	Typical	97	6.3	Reference	Reference
	Atypical	99	3.7	0.66 (0.50–0.87)^‡^	0.70 (0.53–0.93)^†^
CHD	Typical	867	6.3	Reference	Reference
	Atypical	459	5.1	0.80 (0.72–0.90)^‡^	0.85 (0.76–0.96)^‡^
Ventricular arrhythmias	Typical	382	2.8	Reference	Reference
	Atypical	242	2.7	0.94 (0.80–1.10)	0.93 (0.79–1.10)

*P < 0.10, ^†^ *P* < 0.05, ^‡^ *P* < 0.01, ^#^Not calculable due to small number of cases.

**Table 7:** Causes of death categorised according to ICD-10 chapter in current users of typical and atypical antipsychotics.

Class	Atypical		Typical	
	Events (*N*, %)	Incidence rate per 1000 person-years (95% CI)	Events (*N*, %)	Incidence rate per 1000 person-years (95% CI)
A00-B99: certain infectious and parasitic diseases	63 [0.9]	1.98 (1.52–2.53)	46 [0.5]	1.41 (1.03–1.89)
C00-D89: neoplasms/diseases of the blood and blood forming organs and certain disorders involving the immune mechanism	585 [8.2]	1.68 (1.55–1.83)	3955 [43.1]	4.83 (4.68–4.98)
E00-E90: endocrine, nutritional, and metabolic disorders	114 [1.6]	1.50 (1.24–1.80)	106 [1.2]	1.75 (1.43–2.12)
F00-F99: mental and behavioural disorders	907 [12.7]	1.45 (1.36–1.55)	607 [6.6]	1.49 (1.37–1.61)
G00-G99: diseases of the nervous system	657 [9.2]	1.37 (1.27–1.48)	384 [4.2]	1.41 (1.27–1.55)
I00-I99: diseases of the circulatory system	2119 [29.6]	1.66 (1.59–1.73)	1755 [19.1]	1.72 (1.64–1.81)
I20-I25: ischemic heart disease	670 [9.4]	1.59 (1.48–1.72)	570 [6.2]	1.57 (1.44–1.70)
I50: heart failure	108 [1.5]	1.71 (1.40–2.07)	94 [1.0]	2.11 (1.70–2.58)
J00-J99: diseases of the respiratory system	924 [12.9]	1.52 (1.42–1.62)	819 [8.9]	1.57 (1.46–1.68)
K00-K93: diseases of the digestive system	212 [3.0]	1.33 (1.15–1.52)	191 [2.1]	1.40 (1.21–1.61)
L00-L99: diseases of the skin and subcutaneous tissue	20 [0.3]	2.78 (1.70–4.30)	18 [0.2]	1.22 (0.72–1.93)
M00-M99: diseases of the musculoskeletal system and connective tissue	44 [0.6]	1.83 (1.33–2.45)	34 [0.4]	1.90 (1.32–2.66)
N00-N99: disease of the genitourinary system	183 [2.6]	1.51 (1.30–1.74)	162 [1.8]	1.67 (1.42–1.94)
Q00-Q99: congenital malformations, deformations, and chromosomal abnormalities	8 [0.1]	2.04 (0.88–4.02)	8 [0.1]	2.13 (0.92–4.19)
R00-R99: symptoms, signs, and abnormal clinical and laboratory findings	264 [3.7]	1.58 (1.40–1.79)	265 [2.9]	2.08 (1.83–2.34)
V01-Y98: external causes of morbidity and mortality	277 [3.9]	1.63 (1.44–1.83)	160 [1.7]	1.26 (1.07–1.47)

**Table 8:** Relative risk of all-cause mortality (excluding suicide), cardiac mortality, and sudden cardiac death in users of antipsychotics compared to psychiatric nonuser controls stratified by age and daily dose.

Outcome	Characteristic	No. of cases	Incidence rate per 1000 person-years	Fully adjusted RR (aRR) (95% CI)
All-cause mortality	Age < 30	121	4.5	4.12 (2.72–6.25)^‡^
	Age 30–64	3,473	22.3	2.23 (1.85–2.68)^‡^
	Age 65–79	6,563	127.6	1.78 (1.57–2.02)^‡^
	Age ≥ 80	13,684	322.3	1.43 (1.31–1.55)^‡^

Cardiac mortality	Age < 30	7	0.4	—(NC^#^)
	Age 30–64	219	2.8	2.39 (1.80–3.19)^‡^
	Age 65–79	682	27.1	1.60 (1.40–1.83)^‡^
	Age ≥ 80	1,570	63.0	1.55 (1.22–1.98)^‡^

SCD-primary definition	Age < 30	1	0.5	—(NC^#^)
	Age 30–64	8	1.0	—(NC^#^)
	Age 65–79	12	4.5	—(NC^#^)
	Age ≥ 80	23	7.4	—(NC^#^)

All-cause mortality	Low daily dose	21208	97.0	1.72 (1.62–1.84)^‡^
	Medium daily dose	1978	52.5	1.94 (1.80–2.10)^‡^
	High daily dose	655	31.9	1.89 (1.71–2.09)^‡^

Cardiac mortality	Low daily dose	2157	20.0	1.65 (1.36–2.00)^‡^
	Medium daily dose	251	9.9	2.10 (1.68–2.63)^‡^
	High daily dose	70	5.6	2.27 (1.68–3.07)^‡^

SCD-primary definition	Low daily dose	39	3.3	5.87 (2.92–11.78)^‡^
	Medium daily dose	4	1.4	5.51 (1.69–17.96)^‡^
	High daily dose	1	0.8	3.81 (0.47–30.59)

*P < 0.10, ^†^ *P* < 0.05, ^‡^ *P* < 0.01, ^#^Not calculable due to small number of cases.
